# Circadian Influences on Brain Lipid Metabolism and Neurodegenerative Diseases

**DOI:** 10.3390/metabo14120723

**Published:** 2024-12-22

**Authors:** Yusuf Hussain, Mohammad Irfan Dar, Xiaoyue Pan

**Affiliations:** 1 Department of Foundations of Medicine, New York University Grossman Long Island School of Medicine, Mineola, NY 11501, USA; yusuf.hussain@nyulangone.org (Y.H.);; 2 Diabetes and Obesity Research Center, NYU Langone Hospital-Long Island, Mineola, NY 11501, USA

**Keywords:** circadian rhythm, lipid metabolism, Alzheimer’s disease, brain function, fatty acid, cholesterol

## Abstract

Circadian rhythms are intrinsic, 24 h cycles that regulate key physiological, mental, and behavioral processes, including sleep–wake cycles, hormone secretion, and metabolism. These rhythms are controlled by the brain’s suprachiasmatic nucleus, which synchronizes with environmental signals, such as light and temperature, and consequently maintains alignment with the day–night cycle. Molecular feedback loops, driven by core circadian “clock genes”, such as Clock, Bmal1, Per, and Cry, are essential for rhythmic gene expression; disruptions in these feedback loops are associated with various health issues. Dysregulated lipid metabolism in the brain has been implicated in the pathogenesis of neurological disorders by contributing to oxidative stress, neuroinflammation, and synaptic dysfunction, as observed in conditions such as Alzheimer’s and Parkinson’s diseases. Disruptions in circadian gene expression have been shown to perturb lipid regulatory mechanisms in the brain, thereby triggering neuroinflammatory responses and oxidative damage. This review synthesizes current insights into the interconnections between circadian rhythms and lipid metabolism, with a focus on their roles in neurological health and disease. It further examines how the desynchronization of circadian genes affects lipid metabolism and explores the potential mechanisms through which disrupted circadian signaling might contribute to the pathophysiology of neurodegenerative disorders.

## 1. Introduction

In mammals, circadian rhythms are natural, internal processes that regulate various physiological, mental, and behavioral cycles within an approximately 24 h period. These rhythms are driven by a biological clock, located primarily in the brain’s suprachiasmatic nucleus (SCN) in mammals, which synchronizes with environmental cues, such as light and temperature, thereby maintaining alignment with day–night cycles. Circadian rhythms influence critical bodily functions, including the sleep–wake cycle, blood pressure, body temperature, energy metabolism, hormone release, motor activity, and immune function [1,2,3]. They are governed by complex molecular feedback loops involving “clock genes”, which generate rhythmic patterns of gene expression and protein production in central or peripheral systems. Disruptions in circadian rhythms—often caused by irregular sleep, shift work, social jet-lag, travel across time zones, nocturnal light exposure, or lifestyle factors—have been associated with various health issues, such as sleep disorders, mood disorders, metabolic syndromes, obesity, diabetes, fatty liver diseases, cardiovascular diseases (CVDs), infection, changes in the microbiota, cancer, aging, and neurodegenerative diseases, thus underscoring the importance of these rhythms in maintaining overall health and well-being [4,5,6].

The circadian rhythm is regulated via photic and non-photic stimuli. The photic input is provided by intrinsically photosensitive retinal ganglion cells, which send signals through the retinohypothalalmic tract via glutamatergic synapses to neurons in the SCN. This process supports synchronization of the circadian clock [7]. Removal of the SCN disrupts patterns of neuronal activity, causes loss of synchronized circadian rhythms in clock gene oscillations in most tissues, and ultimately results in behavioral and physiological arrhythmicity [8]. The non-photic input to the SCN is derived from various brain regions and is essential for the modulation of circadian rhythms. The SCN expresses several serotonin (5-HT) receptors, and serotonergic input from the midbrain raphe influences the SCN’s response to light by regulating phase shifts in the circadian clock [9]. The intergeniculate nucleus (IGL), another integral part of the circadian rhythm, comprises neurons that express neuropeptide Y (NPY) and gamma-aminobutyric acid (GABA). The IGL projects to the SCN via the geniculohypothalamic tract and facilitates phase shifts in circadian rhythms during the daytime. Stimulation of the medial raphe nucleus elevates serotonin levels within the SCN, whereas activation of the dorsal raphe nucleus increases serotonin concentrations in the IGL; therefore, the IGL aids in synchronizing circadian rhythms to light pulses [10,11]. The response is further propagated by circadian rhythm genes. Key circadian genes, including Clock, Bmal1, Per (Per1, Per2, and Per3), and Cry (Cry1 and Cry2), form a network of feedback loops essential for maintaining rhythmic gene expression [12]. In mammals, these genes are expressed in the SCN and peripheral tissues, and they synchronize bodily functions with external cues, such as light and darkness. The core circadian genes Clock and Bmal1 form a protein complex that activates the Per and Cry genes. The Per and Cry protein products in turn inhibit Clock and Bmal1, thus creating a regulatory loop that keeps time on an approximately 24 h cycle; this process occurs through a transcriptional and translational feedback loop [13,14]. Disruptions in circadian gene expression are associated with various health conditions, such as cardiovascular diseases, cancer, immune-related diseases, and metabolic and neurodegenerative diseases [15,16].

Lipids are essential for the structural integrity and fluidity of neuronal cell membranes by supporting synaptic activity and efficient neurotransmitter release. Alterations in lipid composition can disrupt these functions, impair neural communication, and contribute to cognitive deficits, as observed in Alzheimer’s disease (AD) and Parkinson’s disease (PD) [17]. Additionally, lipids form a substantial part of myelin, an insulating layer around nerve fibers that is crucial for rapid signal transmission; consequently, disruptions in lipid balance can compromise myelination. This demyelination contributes to diseases such as multiple sclerosis, which are characterized by impaired nerve conduction, and symptoms such as muscle weakness and cognitive decline [18]. Lipid metabolism also produces bioactive molecules that regulate inflammation; however, when dysregulated, these processes can lead to excessive neuroinflammation [19,20]. This chronic inflammation, driven by lipid-derived mediators, is associated with neurodegenerative diseases and exacerbates neuronal damage. The brain’s lipid-rich environment makes it vulnerable to oxidative stress, and dysregulated lipid metabolism can lead to lipid peroxidation and the production of toxic by-products that damage neuronal cell structures and contribute to neurodegeneration in diseases [21]. Furthermore, cholesterol, an essential lipid in neurons, regulates protein processing; abnormalities in cholesterol metabolism can lead to the accumulation of amyloid-beta (Aβ) plaques, a hallmark of AD [22]. Genetic disorders that impair lipid metabolism, such as Niemann–Pick or Gaucher disease, cause lipid accumulation in neurons, which in turn triggers cell death and neurodegenerative symptoms [23]. Potential therapeutic strategies, including lipid-lowering drugs and modulation of lipid-associated receptors, are under exploration to target neuroinflammation and oxidative stress in these conditions, thus underscoring the critical roles of lipid metabolism in brain health and the pathogenesis of neurological disorders.

Brain immune cells such as microglia follow circadian patterns, and disrupted circadian signaling can lead to dysregulated microglial activity and consequently increase inflammatory responses that further impair lipid metabolism and promote neurodegeneration [24]. Overall, a well-functioning circadian rhythm is essential for lipid balance in the brain, whereas its disruption can initiate or exacerbate pathological processes associated with neurological disorders. Herein, we discuss the relationships among circadian rhythm, lipid metabolism, and neurological disorders. In addition, we explain how the desynchronization of circadian genes deregulates lipid metabolism in the brain and leads to the development of neurological disorders.

## 2. Mechanisms and Physiological Roles of Circadian Genes

The circadian rhythm generated by the SCN operates through a complex mechanism involving delayed negative feedback within a core transcriptional feedback loop. This system, referred to as the transcription translation oscillating (TTO) loop, is crucial for sustaining rhythmic biological functions across approximately 24 h cycles [13,25]. Within this framework, the core circadian proteins CLOCK and BMAL1 play critical roles. These proteins form heterodimers that bind specific DNA sequences (such as CACGTG), known as E-box response elements, in the promoters of target genes, and subsequently initiate the positive transcriptional phase of the TTO loop and drive the expression of various clock genes [26]. Key target genes regulated by this process include the Period (Per) genes (PER1, PER2, and PER3) and the Cryptochrome (Cry) genes (CRY1 and CRY2). After transcription, the proteins products of these genes, PER and CRY, accumulate within the cell and translocate into the nucleus, where they interact with the CLOCK/BMAL1 complex. This interaction inhibits CLOCK and BMAL1 activity and promotes the transcriptional repression phase of the TTO loop, a negative feedback mechanism essential for generating the oscillations characteristic of circadian rhythms. Beyond CLOCK and BMAL1, accessory proteins such as D-site albumin-binding protein (DBP) and nuclear orphan receptors, including retinoid-related orphan receptor (ROR) and REV-ERBα, as clock-controlled genes, contribute to the regulation of the TTO loop [27]. CLOCK/BMAL1 activates the transcription of REV-ERBα and RORα, which influence BMAL1 gene transcription through another feedback loop and add a further mode of regulation to the system. ROR enhances, whereas REV-ERBα represses, Bmal1 expression, thereby fine-tuning the oscillatory behavior of the circadian clock [28,29]. Recent studies have emphasized the complexity of circadian regulation by demonstrating the importance of additional transcriptional loops; for instance, PER proteins repress their own transcription as well as that of CRY1 and CRY2, thus ensuring synchronization with environmental cues by adding another layer of regulation to the circadian clock [30,31,32]. DEC1 (BHLHe40) and DEC2 (BHLHe41) are transcription factors belonging to the Basic Helix–Loop–Helix (bHLH) protein superfamily, which includes other core clock proteins such as CLOCK and BMAL1. Both DEC1 and DEC2 are regulated by binding of the CLOCK and BMAL1 heterodimer to the E-box or E-box-like sequences in their gene promoters. Once expressed, DEC1 and DEC2 act as negative regulators by competing with CLOCK for DNA binding, thereby inhibiting their own transcription [33]. This negative feedback loop acts in concert with the CLOCK/BMAL1 loop, which involves other negative regulators (PER and CRY), thus maintaining the circadian expression of DEC1 and DEC2 [34].

**Figure 1:**
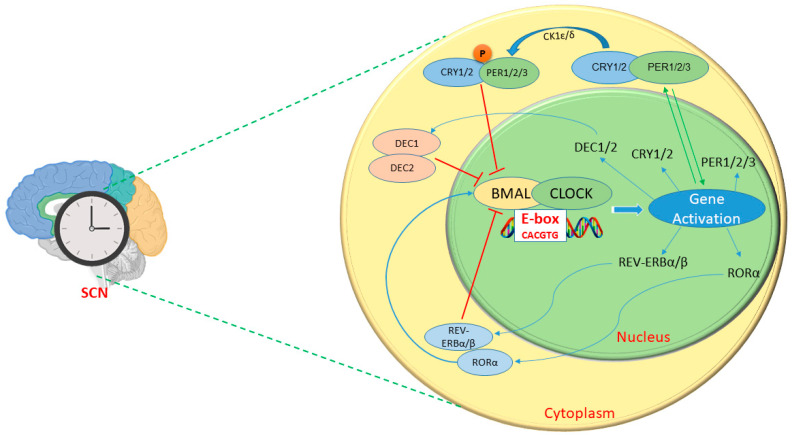
Circadian clock gene regulation in brain. The central circadian clock in the SCN regulates body rhythms and sends signals to peripheral clocks. The CLOCK/BMAL1 complex binds E-box elements on target genes (CRY1/2, PER1/2/3, REV-ERBα/β, and RORα). PER and CRY proteins form cytoplasmic heterodimers that shuttle to the nucleus. After phosphorylation by CK1δ and CK1ε, PER/CRY suppress E-box gene transcription via CLOCK/BMAL1.

Notably, advancements in research techniques, such as global chromatin immunoprecipitation followed by sequencing (ChIP-Seq), have elucidated DNA-binding interactions of core clock proteins and revealed “CLOCK-independent” interactions involving PER and CRY proteins [35]. These interactions occur independently of the CLOCK/BMAL1 complex yet remain time-dependent; therefore, additional mechanisms beyond the primary feedback loop might govern circadian rhythms. Moreover, the roles of post-translational modifications in regulating the circadian clock are increasingly being recognized. The phosphorylation of PER proteins by casein kinase−1 δ/ε (CK1δ/ε) is critical for their nuclear translocation and enables their function in the TTO loop [36,37]. In the nucleus, PER proteins inhibit their own transcription and that of CRY genes, thus effectively closing the feedback loop. By contrast, protein phosphatase 1 (PP1) interacts with PER1, and is likely to remove phosphate groups from PER1 and to promote its cytoplasmic localization [30]. This process decreases PER1 levels in the nucleus, thereby influencing the overall circadian rhythm. The interplay between phosphorylation by CK1δ/ε and dephosphorylation by PP1 is essential for the proper regulation of PER proteins and consequently the maintenance of circadian rhythms [38,39]. A recent study of intracranial Electroencephalography (EEG) data from 38 patients found that brain pathology reduces circadian and ultradian rhythms across regions, independent of event frequency or severity, which highlights a strong link between brain pathology and circadian rhythm disruptions [40]. This dynamic balance is fundamental in ensuring that the circadian clock accurately responds to environmental cues and maintains physiological homeostasis. A brief mechanism is depicted in Figure 1.

In summary, the circadian clock is a highly intricate system involving multiple proteins, feedback loops, and regulatory mechanisms. Understanding these molecular interactions and modifications not only enhances knowledge of circadian biology but also opens avenues for potential therapeutic interventions in disorders associated with circadian rhythm disruptions, such as sleep disorders, metabolic syndrome, and neurodegenerative diseases.

## 3. Circadian Genes

### 3.1. BMAL1/CLOCK

Developmentally, Basic Helix–Loop–Helix ARNT-Like 1 (Bmal1, also called Arntl) is enriched in the cortex perinatally, where it influences neuronal migration and axonal projections, and is regulated by factors such as Glu, Ca^2+^, and protein kinases [41,42,43,44,45]. In adult brains, Bmal1 expression varies across regions and cell types, and shows notable enrichment in Purkinje cells and certain interneurons, such as PV-positive neurons in the prefrontal cortex [46]. Bmal1′s co-expression with neuropeptides, such as substance P and enkephalin, in many forebrain neurons suggests its neurogenesis role in the brain. Gene-targeting studies have shown that BMAL1 deletion or knockdown leads to circadian and behavioral abnormalities, as well as phenotypes resembling psychiatric and neurodegenerative conditions [44,47,48]; therefore, Bmal1 may have a critical role in both circadian regulation and brain health. Several peripheral-tissue-specific Bmal1-deficient mouse models have been shown to control lipid metabolism homeostasis [49,50,51] and atherosclerosis [52,53,54]. Kress et al. have shown that global Bmal1 deletion disrupts Aβ rhythmicity in hippocampal interstitial fluid (ISF) [55]. However, hippocampus-specific Bmal1-deficient mice do not show marked effects on Aβ levels or hippocampal amyloid pathology. In addition, Nestin-Cre+/Bmal1(f/f) mice have Bmal1 deletion in neurons and astrocytes but not the central SCN and show no effects on hippocampal ISF Aβ rhythm [55]. These studies suggest that ISF Aβ rhythms are mediated by central clocks in the SCN and transmitted to the hippocampus. However, Kress et al. have found that knockout or KD of Bmal1 directly induces local apolipoprotein E (ApoE) gene expression [55]. Chen et al. have identified that BMAL1 in rs2278749 T/C polymorphism is associated with high AD risk in ApoE4-negative people [56]. Therefore, a strong biological rationale exists for Bmal1 deficiency leading to AD-associated circadian disorder.

The circadian rhythm gene BMAL1 also affects adipogenesis and overall energy homeostasis. In individuals with metabolic syndrome, dysregulated BMAL1 function in adipose tissue leads to imbalances in metabolic processes (such as alterations in NADPH levels, mitochondrial electron transport, diurnal activation of UCP1, and inhibition of NF-κB, ultimately disrupting normal energy balance) [57,58,59]. A lack of BMAL1 function, as observed in Bmal1-deficient mice, increases circulating fatty acids (FAs), including triglycerides (TGs) and free FA (FFA) [60]. This elevation indicates decreased fat storage capacity in adipose tissue, which in turn leads to ectopic fat deposition in organs. The absence of ectopic fat formation in tissue-specific Bmal1 knockout mice suggests that the regulation of Bmal1 is essential for managing fat storage and energy utilization across the body [60]. Collectively, these findings highlight the integral role of circadian rhythms, particularly through Bmal1, in maintaining metabolic homeostasis, and further demonstrate the potential consequences of Bmal1 dysfunction, including metabolic syndrome and related disorders. Understanding these connections may provide insights into how circadian rhythms influence energy metabolism, lipid metabolism, and overall brain health. Therefore, further research is necessary in this area.

In mammals, as a Bmal1 heterodimeric, Clock might play an important role in brain development and plasticity. Clock mutant (*Clock*Δ19) mice develop a metabolic syndrome with high cholesterol and TG in circulation [61]. We have demonstrated that the mutant Clock gene increases atherosclerosis and induces fatty liver diseases through cholesterol efflux and FA uptake pathways in macrophages and the liver, respectively [62,63]. Several studies have shown that Clock-Δ19 mice have altered dopaminergic activity and increased expression and phosphorylation of tyrosine hydroxylase dopaminergic transmission [64,65,66]. However, the role of the Clock in the brain’s lipid metabolism remains unclear.

### 3.2. PER (PER1, PER2, and PER3)

Period Circadian Regulator genes (PER1, PER2, and PER3) are expressed primarily in the central nervous system (CNS), including the SCN of hypothalamus and peripheral nervous systems, and play essential roles in maintaining circadian rhythms that regulate physiological processes such as sleep–wake cycles and hormone production. Per1 is critical for generating and maintaining circadian rhythms in the SCN and is highly responsive to light, thus functioning in resetting the circadian clock in response to environmental changes. The limited sequence similarity between mouse Per1 and Per2 (73.4% at the amino acid level) leads to distinct, non-overlapping functions. Mutations in Per1 can alter sleep patterns and shorten circadian periods. Per2 has a more dominant role in rhythm stability by regulating Per1 and other clock genes through feedback loops essential for circadian periodicity; Per2 mutations often shorten circadian rhythms and make them more susceptible to environmental cues such as constant darkness [67]. Unlike Per1 and Per2, Per3 has a limited role in the core circadian clock, but it influences sleep–wake patterns and homeostasis. PER3 polymorphisms are associated with sleep preferences, mood disorders, and cognitive performance under sleep deprivation, and its variable number tandem repeat is associated with sleep and psychiatric conditions [5,68]. Per1 therefore initiates and maintains rhythms responsive to environmental cues [69], Per2 stabilizes and regulates gene expression rhythms, and Per3 influences sleep and peripheral circadian processes [70]. Per2 is therefore central to circadian regulation, whereas Per1 and Per3 contribute to specific functions such as light responsiveness and sleep behavior and together support the body’s internal clock [71]. In turn, the master circadian clock is regulated not only by the brain but also by food intake, which has been found to regulate central circadian clock genes [72]. Several studies have shown that time-restricted feeding (TRF) schedules regulate Per1, Per2, and Per3 in rat SCN and the paraventricular and arcuate (ARC) hypothalamic nuclei [72,73,74]. While their roles in neuronal development are not fully clear, studies suggest their involvement in neural dysfunction; for example, in one of the studies it was found that the loss of dopaminergic neurons may result from a null mutation in the Per gene, promotes premature animal mortality when combined with short-term oxidative stress, and worsens the age-dependent loss of dopaminergic neurons [75]. In another study in particular, *Per1^−/−^* mice show reduced autophagy and greater neuronal vulnerability to ischemic damage [76]. These suggest that Per plays an important role in regulating brain function.

### 3.3. CRY (CRY1 and CRY2)

In mammals, the circadian cryptochromes are CRY1 and CRY2. CRY1 is sensitive to light, which influences its stability and activity [77]. Light exposure can lead to the degradation of CRY1 and consequently allow the transcriptional activation of clock genes to resume. This process is crucial for synchronizing internal circadian rhythms with the external light–dark cycle. Disruption or mutations in the CRY1 gene can alter circadian rhythms and lead to sleep disorders and metabolic dysregulation. CRY1 has been found to play roles in regulating sleep patterns, metabolism, and stress responses [78].

Similarly to CRY1, CRY2 functions as a negative regulator in the circadian clock by inhibiting the transcriptional activity of CLOCK and BMAL1 and contributing to the feedback mechanism regulating PER gene expression. The finding that mutations in CRY2 can lead to longer circadian periods suggests essential functions of CRY2 in maintaining the proper timing of biological rhythms. CRY2 is involved in regulating sleep–wake cycles. Its expression levels fluctuate throughout the day, and alterations in its function can influence sleep quality and duration. CRY2 interacts with various proteins, including REV-ERBα, which can also influence the circadian rhythm, thus underscoring its multifaceted role in clock regulation [12]. CRY2 has been implicated in metabolic processes and is associated with mood disorders. Its disruption may contribute to metabolic syndrome and psychiatric conditions, including depression and anxiety [79]. Several studies have found that the knockout of Cry1 mice directly altered activity levels throughout the day, displayed abnormal electrical activity, and altered gene expression in the brain [80,81,82]. Several studies have suggested cryptochrome (Cry1 and Cry2) genes are associated with cognitive function and anxiety-related behaviors [82,83,84]. Thus, Cry1 knockout or Cry2 knockout mice are good models for studying the underlying mechanisms of circadian sleep disorders in humans.

### 3.4. DEC (DEC1 and DEC2)

DEC1 (Basic Helix–Loop–Helix Family Member E40, also called BHLHE40) is responsive to light signals, and its expression is induced by light pulses. Consequently, DEC1 aids in resetting or adjusting the circadian phase of the central clock located in the SCN, the brain’s master circadian pacemaker. In peripheral tissues, DEC1 expression is upregulated by TGF-β, which in turn resets the circadian phase of peripheral clocks in those tissues [85]. These findings highlight DEC1’s role in synchronizing peripheral clocks with external cues and central signals. Both DEC1 and DEC2 influence circadian rhythms beyond the molecular clock by modulating clock output signals that regulate behavior and metabolism, thereby ensuring that circadian rhythms are properly aligned with physiological processes. This interaction between DEC1/DEC2 and CLOCK/BMAL1 adds a further layer of complexity to the overall regulation of circadian rhythms in both central and peripheral systems. The feedback loop between the CLOCK/BMAL1 dimer and the DEC1/PER or DEC1/CRY dimers underpins the oscillations in gene expression that manifest as the body’s circadian rhythm. These rhythmic cycles regulate many biological processes, including sleep–wake cycles, metabolism, and hormone secretion. One study has reported that DEC1 and DEC2 exhibit consistent rhythmicity and phase synchrony across multiple brain regions in control participants, whereas this rhythmic pattern is notably weaker in patients with major depressive disorder [86]. That study suggests that the disruption of circadian gene (DEC1 and DEC2) expression in major depressive disorder may affect regulating mood; consequently, DEC1 and DEC2 might serve as potential molecular targets for mood disorder treatments [86]. DEC1 suppresses PPARγ target genes, which are crucial for lipid metabolism, in white adipose tissue. DEC1 deficiency disrupts the circadian rhythm of PPARγ target genes, and consequently increases nighttime expression and decreases serum free FA levels [87,88]. Although DEC1 and DEC2 are well known for regulating circadian rhythms and lipid metabolism, they have not been extensively studied in neurological disorders.

## 4. Lipid Composition in the Brain

Lipids and their intermediates are fundamental to brain architecture and function and make up approximately 50% of the brain’s dry weight. The brain is the second most lipid-rich organ after adipose tissue. Unlike adipose tissue, which stores FAs predominantly as triglyceride (TG), the brain uses acylated lipids primarily for the biosynthesis of phospholipids, which are essential for cell membrane integrity. The brain’s lipid profile is characterized by high levels of long-chain polyunsaturated FAs, including arachidonic acid, eicosapentaenoic acid, and docosahexaenoic acid (DHA). Whereas some FAs are synthesized de novo, essential FAs are transported from the systemic circulation across the blood–brain barrier (BBB) [89]. Recent findings suggest an active dynamic process in which as much as 8% of long-chain polyunsaturated FAs undergo daily turnover and replacement by plasma-derived FAs [90]. Radiolabeling studies have demonstrated that FAs cross the BBB and are incorporated into neuronal phospholipids. For instance, radiolabeled FAs introduced into the carotid artery have been detected in neuronal cells [91], and brain perfusion studies show rapid incorporation of palmitate into cerebral phospholipids, regardless of delivery via plasma, apolipoprotein-containing plasma, or albumin-containing synthetic saline [92,93]. These findings suggest albumin’s role in facilitating FA transport, although the exact transport mechanisms remain to be elucidated.

**Figure 2:**
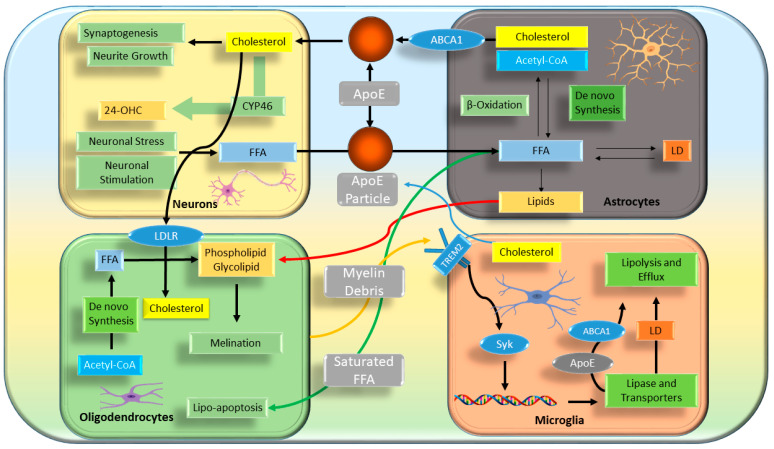
Lipid trafficking in the brain. Astrocytes synthesize cholesterol via HMG-CoA reductase (HMG-CoAR), package it into ApoE lipoproteins, and export it via ABCA1. Neurons receive APOE/C1 lipoprotein for neurite growth, synaptogenesis, or conversion to 24-OHC by cholesterol 24-hydroxylase (CYP46). Stimulated or stressed neurons release FAs in APOE particle lipoprotein to astrocytes for degradation or storage in lipid droplets. Lipids for oligodendrocyte myelination/remyelination are synthesized by both astrocytes and oligodendrocytes, and ApoE aids in astrocyte-to-oligodendrocyte transport. Also, neurons can transfer cholesterol-rich lipoproteins to oligodendrocytes through low-density lipoprotein receptor (LDLr) on oligodendrocytes. Excess saturated FAs from astrocytes can cause oligodendrocyte death via lipoapoptosis. Lipids from myelin debris activate triggering receptor expressed on myeloid cells 2 (also called TREM2) signaling. TREM2 expression triggers spleen tyrosine kinase (Syk) activation, triggering lipid metabolism genes that aid in lipid droplet breakdown and lipid efflux, and is also known to participate in neurological disorders [97,98,99,100,101].

The precise mechanism through which FAs traverse the BBB remains an unresolved and fundamental question in neurobiology. Whereas passive diffusion of FAs across the BBB cannot be entirely excluded, emerging evidence underscores the involvement of specific FA transport proteins in mediating this process. Expression studies in humans and mice have identified membrane-localized FA transport proteins (FATP1 and FATP4) as the predominant transporters at the BBB, whereas FA translocase/CD36 has been implicated in facilitating FA translocation across human brain microvascular endothelial cells [94]. Additionally, cytosolic FA-binding protein 5 (FABP5) plays a critical role in FA uptake within cultured brain microvascular cells. Growing evidence suggests that certain transporters may exhibit substrate specificity [95]. For instance, the major facilitator superfamily domain-containing protein 2a (Mfsd2a), expressed exclusively in the endothelium of the BBB, has been demonstrated to selectively transport DHA in the form of lysophosphatidylcholine [96]. In brief, lipid transportation is described in Figure 2 on the basis of previous findings [97,98]. This specificity highlights the complexity of FA transport mechanisms across the BBB, which remains an area of active investigation.

### 4.1. Cholesterol in the Brain

Cholesterol, because of its large size, cannot penetrate the BBB and instead reaches the brain in the form of 24-hydroxycholesterol (HC) and 27-HC [102,103]. Brain cholesterol metabolism operates independently of that in peripheral tissues. In the brain, cholesterol has a long half-life and is efficiently recycled. Brain cholesterol is synthesized primarily de novo in glial cells, particularly astrocytes, whereas neurons rely on cholesterol supplied by astrocytes for their function. The transport of cholesterol occurs through ApoE and ApoC1 [92], which are loaded with cholesterol in astrocytes and transported via ATP-binding cassette (ABC) transporters. Neurons take up these lipidated ApoE particles through receptor-mediated endocytosis via low-density lipoprotein receptors (LDLRs) and LDLR-related protein 1 (LRP1) [104]. Although oligodendrocytes can also synthesize cholesterol, they acquire it from astrocytes via lipoprotein particles [97]. Deficiencies in neuronal cholesterol can severely impair essential processes such as synaptic vesicle exocytosis, neuronal signaling, and neurotransmission, and lead to synaptic degradation and neurodegeneration [105,106]. Disruptions in CNS cholesterol regulation are associated with various neurological disorders, including Niemann–Pick type C (NPC) disease and Parkinson’s disease [107]. Astrocytes play crucial roles as a primary source of lipoprotein synthesis in the brain by producing ApoE and ApoC1 at levels second only to those produced in the liver. The high-density lipoprotein (HDL)-like lipoproteins secreted by astrocytes are crucial for neuronal health, because neurons use LDLRs to absorb these lipoproteins, thus facilitating lipid transfer essential for axonal growth and neuronal survival. Beyond lipid metabolism, ApoC1 and ApoE serve as ligands for various neuronal receptors and consequently influence multiple physiological processes [108].

Cholesterol efflux in the brain is regulated by membrane transporters such as ABCA1 and ABCG4, which are integral to the formation of cholesterol-rich ApoE particles. Total Abca1 deficiency in mice modestly decreases brain cholesterol levels, and results in slight impairments in neurite morphology and memory [109,110]. ATP-Binding Cassette Sub-Family A Member 7 (ABCA7) also plays a crucial role in cholesterol homeostasis in the brain. ABCA7 assists in moving cholesterol out of microglia or neurons to HDL particles and releasing it into the circulation [111]. Abca7 deficiency induces ER stress in neurons and activates the SREBP2-BACE1 pathway [112], thereby contributing to AD pathogenesis. Similarly, findings that deficiencies in ABCG1 and ABCG4 correlate with decreased cholesterol content and memory deficits underscore the critical role of astrocytic cholesterol metabolism in maintaining a proper neuronal cholesterol supply essential for learning and memory [113,114]. Cholesterol oxidation products are cytotoxic, and both excessive and insufficient cholesterol can lead to cell death, increased endoplasmic reticulum stress, and cognitive decline [115].

Cholesterol also plays a critical role in synaptic transmission by supplying presynaptic and postsynaptic membrane structures. Abnormal cholesterol levels can disrupt neurotransmission, as evidenced by studies showing that a high-cholesterol diet in rats increases brain cholesterol, TG, and LDL-C, and leads to neurotransmitter imbalances characterized by elevated glutamate and dopamine but diminished gamma-aminobutyric acid and serotonin levels [116]. Mice on high-cholesterol diets exhibit psychomotor impairments and depressive behaviors. Additionally, LDLR knockout studies have indicated that cholesterol influences acetylcholinesterase activity, whereas cholesterol depletion weakens N-methyl-D-aspartate (NMDA) and α-amino-3-hydroxy-5-methyl-4-isoxazolepropionic acid (AMPA) receptor-mediated synaptic currents, thereby affecting neurotransmission and receptor stability [117]. Collectively, these findings highlight the critical importance of cholesterol metabolism in the brain, and its profound implications in neuronal function and overall brain health. Nonetheless, very limited information is available regarding cholesterol regulation on circadian rhythms in the brain.

### 4.2. Fatty Acids in the Brain

Neuronal FA uptake remains poorly understood but is likely to involve FA transporters after BBB translocation. Neurons in the ventromedial hypothalamus (VMH) express FATP1 and CD36, both of which have been implicated in FA uptake. Studies using fura-2 calcium imaging and membrane potential assays have shown that VMH and ARC neurons respond to oleic acid (OA, C18:1 n-9), whereas this response is abolished after CD36 knockdown via adeno-associated viral vectors. CD36, a known lipid sensor, may work alongside other sensors such as GPR120, which is active in hypothalamic neurons and mediates DHA’s anti-inflammatory effects, although its role in neuronal lipid sensing in vivo is unconfirmed. Additionally, FABP3 selectively facilitates neuronal uptake of arachidonic acid but not palmitic acid (C16:0); these findings indicate its specificity in brain FA trafficking [118,119]. Neurons are increasingly being recognized to receive metabolic support from glial cells, particularly astrocytes. The “astrocyte-neuron lactate shuttle” model suggests that astrocytes metabolize glucose to lactate, which in turn is taken up by neurons via monocarboxylate transporters and used as an energy source [120,121]. Astrocytes are also key in FA metabolism, because FA oxidation occurs primarily in astrocytes rather than neurons [122]. High-fat diets increase astrocyte-mediated lipid oxidation and brain ketone levels, thus modulating energy homeostasis in hypothalamic regions such as the VMH and ARC [123]. Hypothalamic astrocytes express fatty acid transporters such as FATP1, FATP4, and CD36. These cells show elevated levels of long-chain fatty acid oxidation by carnitine palmitoyltransferase-1 (CPT1) [124,125]. This oxidation process is regulated by AMP-activated protein kinase (AMPK). Moreover, fatty acid-binding protein 7 (FABP7) is critical for astrocyte-neuron lipid homeostasis, and its deletion leads to neuropsychiatric disorders such as schizophrenia, probably because of altered dendritic spine morphology. Astrocyte transplantation into FABP7 knockout mice partially rescues cognitive deficits [126]. Within cells, long-chain fatty acids (LCFAs) are esterified by acyl-CoA-binding protein (ACBP). This esterification assists hypothalamic astrocytes in sensing LCFAs [126,127]. These findings highlight the complex network of FA transporters, lipid sensors, and cellular interactions that enable precise FA uptake and energy regulation in the brain, particularly within hypothalamic neurons and astrocytes.

### 4.3. Triglycerides in the Brain

Triglycerides (TGs), simple lipids that store and transport energy, come from two main sources: the gut and the liver. More than 6000 TG types exist, on the basis of various FA combinations. TGs are key components of TG-rich lipoproteins (TRLs), such as VLDL, chylomicrons, and their breakdown products. In humans, postprandial TG responses fluctuate throughout the day, and higher levels are observed during the resting phase than the active phase, thus suggesting potential roles of the SCN or daily behaviors [128]. Additionally, epidemiological research has indicated that night-shift workers, who have nighttime activity and meal schedules, have elevated risk of cardiovascular disease [129]. Studies have detected TGs in human cerebrospinal fluid and shown that TGs can cross the BBB [130]. Postprandial TG levels modulate brain responses to food cues in people with genetically decreased dopamine D2 receptor signaling [131]. That study identified a mechanism through which dietary TGs alter reward circuit signaling, thereby linking energy-rich diets to dopamine signaling adaptations that might underlie reward deficits and compulsive behaviors. Animal studies have suggested that high TG levels may impair memory by affecting brain function [128,132]; therefore, lowering TG might help reverse cognitive decline. Another report has suggested improved cognition after TG lowering with gemfibrozil treatment [128]. Lee et al. have demonstrated that lipolytic products of TG-rich lipoproteins enhance the BBB transfer coefficient and cause lipid accumulation in astrocytes [133]; therefore, elevated blood TG might affect intracellular lipid droplet (LD) formation both peripherally and centrally. Brain energy deficits are associated with neurodegenerative disease progression, and nutritional deficiencies and hypoxia are common stressors. Astrocyte exposure to various nutritional stressors—nutrient deprivation, excess free FAs, or L-lactate—for 24 h significantly increases LD size and number [133]. In line with findings from Nguyen et al., DGAT1 and DGAT2 inhibitors decrease LD accumulation in nutrient-deprived astrocytes [134]. Additionally, hypoxia and elevated norepinephrine selectively promote LD accumulation in astrocytes by upregulating glycogenolysis, aerobic glycolysis, and lactate production. Defects in lipolysis, such as those due to Atgl deficiency, also contribute to lipid aggregation, as observed in the accumulation of TG-rich LDs in *Atgl^−/−^* macrophages [135].

## 5. Circadian Rhythm of Lipid Metabolism-Associated Genes in the SCN

Several lipid metabolism-associated genes, such as ABC (ABCA1, ABCA7, ABCG1, and ABCG4) transporters, CD36, NPC1L1, NPC1, MTTP, and DGAT1/2, have been detected in the SCN by SCN-seq [136], and are well known for their relationships with either lipid metabolism or neuronal disorders. These genes show similar rhythmic activities over a 24 h cycle. Among them, Cd36, Dgat1, Dgat2, Npc1l1, Npc1, and Abcg1 exhibit the highest activity in the daytime (the inactive phase) in the brains of mice [136,137].

### 5.1. ABC Transporters

Several ABC transporters (such as ABCA1, ABCA7, ABCG1, and ABCG4) are expressed primarily in the CNS and play essential roles in regulating lipid metabolism and sterol trafficking in the brain. ABCA1 primarily facilitates the efflux of lipids, such as cholesterol and phospholipids, to lipid-free lipoproteins, and ApoE is its main substrate. This process is crucial for maintaining lipid homeostasis; loss-of-function mutations in the ABCA1 gene lead to decreased ApoE levels, which are associated with increased AD risk because of impaired lipid transport and metabolism [138]. Exome sequencing has indicated a significant association between the rates of predicted disruptive ABCA1 variants and AD risk [139]. Similarly, ABCA7 contributes to the transport of phospholipids; genetic and epigenetic studies have identified various single-nucleotide polymorphisms, alternative splicing variants, and methylation patterns in the ABCA7 gene that result in dysfunction, altered lipid metabolism, and heightened AD risk [140]. ABCA7 mediates phospholipids efflux without affecting the cholesterol balance in peripheral tissues [141]. Iqbal et al. have shown that Abca7 plays a role in the biosynthesis and efflux of sphingomyelin [142], a plasma lipoprotein sphingolipid, in Abca7 knockout mice, without affecting cellular uptake and metabolism, synaptic plasticity, and cognition [112].

Together, ABCA1 and ABCA7 are critical in maintaining the lipid balance in the brain, and their dysfunction significantly influences neurodegenerative processes associated with AD. Additionally, ABCG1 plays a crucial role in cholesterol transport within the brain, particularly in facilitating the transfer of cholesterol from astrocytes to neurons—a process essential for neurite outgrowth and synaptogenesis. In this transport pathway, ABCA1 initiates the release of nascent, ApoE-rich lipoprotein particles from astrocytes, which are then enriched with cholesterol and phospholipids by ABCG1 expressed in both astrocytes and neurons [143]. This process enables the maturation of these particles into HDL-like lipoproteins, which subsequently deliver cholesterol to neurons through interactions with receptors such as those in the LDLR family. Furthermore, ABCG4, expressed in neurons and astrocytes in regions such as the hippocampus and cerebellum, has been implicated in sterol trafficking and is found in ependymal cells and microglial cells. This transporter functions as an efflux pump at the BBB and can export desmosterol and Aβ [144]. The coordinated actions of ABCA1, ABCA7, ABCG1, and ABCG4 are therefore essential in ensuring that neurons receive adequate cholesterol and other sterols to support critical developmental and functional processes in the brain while protecting against neurodegenerative conditions such as AD.

Although specific studies examining the direct effects of ABC transporters on circadian rhythms are limited, the involvement of these proteins in lipid metabolism and transport suggests potential roles in the synchronization and maintenance of circadian processes.

### 5.2. Cluster of Differentiation 36 (CD36)

CD36, a multifunctional membrane protein, is expressed in the brain neurovascular unit including microglia, microvascular endothelial cells, astrocytes, and neurons. In normal brains, Cd36 plays an important role in regulating FA transport across the BBB [145]. CD36 also plays critical roles in the absorption and transport of lipids, particularly free FAs and monoacylglycerols, in the intestines, liver, adipocytes, and macrophages. As a scavenger receptor, CD36 facilitates uptake of various lipid molecules, and substantially contributes to lipid metabolism and homeostasis. Beyond its role in FA absorption, Cd36 works alongside Niemann–Pick C1-like protein (Npc1l1) in mediating free cholesterol uptake, a process crucial for maintaining cellular cholesterol levels and membrane integrity [146,147]. Recent studies have highlighted the influence of circadian rhythms on Cd36 expression and activity. For instance, mice expressing the *Clock^∆19/∆19^* mutant protein, which disrupts normal circadian regulation, show elevated Cd36 and Npc1l1 [62,63,148]. These findings suggest that circadian factors modulate lipid uptake mechanisms in the intestines. The interplay between these proteins and circadian rhythms indicates substantial temporal regulation of lipid absorption and metabolism. This regulation is likely to influence not only the efficiency of nutrient uptake but also the overall metabolic health of the organism. Thus, the role of Cd36 in lipid transport is not only fundamental for normal digestive processes but also is intricately associated with circadian biology [149].

### 5.3. Microsomal Triglyceride Transfer Protein (MTP)

Microsomal TG transfer protein (MTTP or MTP) is recognized primarily for its critical role in lipid metabolism, specifically in the assembly and secretion of lipoproteins such as VLDL in the liver and chylomicrons in the intestines [150]. Additionally, MTP functions as a major cellular protein transferring neutral lipids between membrane vesicles and serves as an essential chaperone in the biosynthesis of ApoB-containing TG-rich lipoproteins [151]. Its importance is underscored by the finding that MTTP gene mutations lead to a loss of lipid transfer activity in patients with abetalipoproteinemia [151,152]. Furthermore, recent findings have suggested that MTP also regulates the biosynthesis of both CD1 glycolipid-presenting molecules and cholesteryl esters [151,153]. We have explored circadian regulation in lipid metabolism, focusing on the effects of diminished Clock activity in (Clock ∆19) *Clock^mt/mt^* mice and a Clock knockdown model [154]. Elevated Mtp expression, associated with diminished Clock activity, indicates Clock’s role in regulating lipid metabolism, in agreement with its functions [154,155]. Furthermore, decreasing expression of Bmal1 and Clock—key components of the circadian positive feedback loop—also increases Mtp; therefore, this loop might limit Mtp expression outside feeding periods [154]. Moreover, decreases in negative loop proteins (Pers and Crys) result in either unchanged or decreased Mtp, thereby indicating a potential interplay between both circadian loops in controlling lipid levels and plasma triglyceride concentrations [156]. Mtp may serve as a target for treating severe family hypercholesterolemia and atherosclerosis without causing liver injury by miRNA 30c [157,158]; these findings suggest important roles of Mtp in regulating lipid metabolic pathways in peripheral tissue. Mtp is also expressed in neurons, and a high expression of Mtp is associated with age and brain tumor stage [151,159]; these studies suggest that Mtp may also play critical roles in neurological health and disease.

### 5.4. Acyl-CoA Acyltransferases (DGAT1 or DGAT2)

Acyl-CoA acyltransferase (DGAT) is an enzyme anchored to the ER membrane that catalyzes the terminal step of TG biosynthesis: esterification of long-chain acyl-CoA esters to diglycerides. DGAT has two isoforms, DGAT-1 and DGAT-2, which are encoded by different genes. DGAT-1 is expressed predominantly in enterocytes of the small intestine, where it mediates the reassembly of TGs from dietary FAs for chylomicron formation during intestinal lipid absorption [160]. By contrast, DGAT-2 is expressed primarily in the liver, adipose tissue, and skin, where it synthesizes triglycerides from de novo free FAs and newly formed diglycerides [161]. Dgat-1 knockout (*Dgat1^−/−^*) mice are viable and show modestly diminished tissue triglyceride levels, whereas Dgat-2 knockout (*Dgat2^−/−^*) mice exhibit profound lipopenia (90% decrease in total body triglyceride), impaired skin barrier function, and neonatal lethality [162]. DGAT1 has been reported to be a therapeutic target in glioblastoma, and blocking DGAT1 in glioblastoma models has been found to prevent lipid droplet formation, initiate cell death in tumors, and notably decrease tumor growth [163]. Moreover, DGAT1 protects glioblastoma (GBM) cells against oxidative stress and regulates lipid levels by storing excess FAs [164]. Although Dgat1 and Dgat2 show circadian rhythm correlation [92]. However, no other studies on neurological disorders and circadian clock have been reported.

### 5.5. NPC Intracellular Cholesterol Transporter (NPC1 or NPC2)

Niemann–Pick disease type C (NPC) disease is a rare lipid storage disorder with varying symptoms, including neurological (ataxia, gaze palsy, and seizures), systemic, and psychiatric (bipolar disorder, psychosis, and depression) effects. Approximately 95% of cases are associated with mutations in the Niemann–Pick Disease, Type C1 (NPC1) gene, and the remainder are due to NPC2 mutations. NPC1 encodes a transmembrane protein, whereas NPC2 encodes a soluble protein; both proteins work together in transporting cholesterol from late endosomes/lysosomes to other cell areas [165]. Most people with NPC disease have mutations in the NPC1 gene that affect cholesterol transport within cells. This disruption causes cholesterol and other lipids to accumulate in cellular compartments and leads to dysfunction and progressive neurodegeneration [166]. NPC disease typically appears in childhood but can also manifest in teenagers and adults, and it often results in early death [23]. The exact mechanism underlying neuronal death in NPC remains unclear.

Lipid buildup, particularly involving sphingosine, may disrupt calcium balance and lysosomal function, whereas lipid accumulation in the lysosomes and mitochondria can lead to oxidative stress. NPC1 dysfunction affects mTORC1 and LC3 signaling, and a low dose of HP-β-cyclodextrin to deplete cholesterol, combined with an autophagy inducer to restore autophagic flux, may offer a promising treatment for NPC1 disease [167]. Although NPC affects other organs, restoration of NPC1 in the CNS prevents neurodegeneration in mice [168]. However, neuron-only restoration is not sufficient to treat neurodegeneration; other brain cells such as astrocytes, microglia, and oligodendrocytes are also required to restore; therefore, NPC1 is also essential in other brain cells. High NPC1 expression in oligodendrocytes and microglia has been observed and is necessary for proper oligodendrocyte maturation and myelin maintenance [169,170].

The *Npc1^−/−^* mouse model, with a loss-of-function mutation in the Npc1 gene, mimics early onset human NPC pathology. These mice show neurodegeneration in key regions such as cerebellar Purkinje cells and thalamic neurons, whereas the cortex and hippocampus are less affected. Mild behavioral issues, such as ataxia and tremors, appear around the age of 6 weeks and worsen by 8 weeks. Severe ataxia, feeding difficulties, and weight loss develop by the age of 10–12 weeks [171]. Research has indicated notable changes in microglia number, morphology, and phagosome content in the cerebellum in *Npc1^nmf164^* mice after weaning, before Purkinje cell degeneration [172]. Cerebellar microglia migrate, proliferate, and differentiate postnatally alongside cerebellar neurons and play key roles in development by clearing apoptotic cells and redundant synapses. Normal cerebellar microglia show higher phagocytic activity and gene expression, indicating a stronger cell clearance function than microglia in other brain regions. *Npc1^−/−^* mice disrupt microglia and synapse development in the cerebellum, leading to behavioral deficits and increased risk of Purkinje cell neurodegeneration [172,173].

In coordination with Npc1, Npc2 binds and solubilizes cholesterol, passing cholesterol to Npc1 in late endosomes and lysosomes; Npc1 then moves cholesterol to other cellular parts in neurons, oligodendrocytes, and astrocytes [174,175]. This process is essential for intracellular lipid transport and cholesterol homeostasis. Dysfunction of Npc2 genes is associated with neurodegenerative disorder and Niemann–Pick disease 2, including motor impairment, psychiatric symptoms, neuroinflammation, cellular dysfunction, seizures, and visuospatial and sensory processing [176]. Mutants in the NPC2 genes cause cholesterol accumulation in lysosomes, leading to lipid storage in cells, particularly in neurons, leading to progressive cell dysfunction and death in the brain. In addition to the trafficking of cholesterol, Npc2 is also associated with metabolism and the transporting of sphingolipids in lysosomes of cells in the brain, liver, and macrophage [175,177,178]. These suggest Npc2 plays a vital role in regulating cholesterol, sphingolipid, and lipid transport in the brain and other tissues. To understand the circadian regulation in NPC diseases, Richardson et al. used Npc1 mutant mice to study wheel-running activity measurement, neuropathology, and clock gene expression; they did not find that extensive cholesterol accumulation in the SCN caused circadian disruption in Npc1 (NIH) mutant mice [179]. Similarly to NPC1, NPC2′s primary role is to facilitate the transport of cholesterol from the lysosome to other cellular compartments, including the endoplasmic reticulum (ER), where it is needed for various functions like membrane formation, hormone synthesis, and signaling pathway [180]. In *Npc2^−/−^* (*Npc2^Gt(LST105)BygNya^*) mice, early signs of NPC disease appear as splenomegaly and cerebellar neuroinflammation at the age of 6 weeks, including Purkinje cell loss. Neurological symptoms like tremors and ataxia emerge and worsen rapidly by 8 weeks. The disease reaches its end-stage, marked by growth retardation, brain atrophy, foam cell accumulation in organs, and severe Purkinje cell degeneration, highlighting the rapid progression of neurodegeneration linked to cholesterol trafficking dysfunction by the age of 12 weeks [181]. This suggested that NPC1 and NPC2 are associated with neurological disorders involving memory and cognition such as PD and AD. However, the dysfunction of the circadian clock in SCN will affect lipid metabolism, and cholesterol homeostasis is still unclear in the NPC disease model. Further research is needed to determine the specific regulation underlying the circadian clock, but the connection between circadian rhythms, lipid metabolism, and neurodegeneration offers a new avenue for understanding how disruptions in the circadian clock might affect conditions such as NPC1 and NPC2.

## 6. Roles of Circadian Genes and Lipid Metabolism in Neurological Disorders

Circadian genes have key roles in regulating lipid metabolism, which in turn affects neurological health. Core circadian genes, such as Clock, Bmal1, Per1, and Cry1, coordinate lipid synthesis, storage, and breakdown in daily cycles. Disruptions in these genes can lead to metabolic imbalances, particularly in brain lipids, which are essential for neuron function and membrane integrity. Altered lipid metabolism is associated with neurodegenerative diseases such as AD and PD by affecting membrane fluidity, signal transduction, and energy homeostasis [156]. Mutations in circadian genes or clock-controlled genes exacerbate lipid dysregulation, thereby increasing oxidative stress and the accumulation of neurotoxic lipid derivatives. For example, deficiencies in lipid-modulating proteins such as sirtuin 1 (SIRT1) in circadian pathways contribute to toxic lipid buildup and promote neuroinflammation and cell damage [182]. Similarly, REV-ERBα is a key node linking circadian and lipogenic pathways through its regulation of SREBP signaling and bile acid homeostasis, both of which are critical in lipid metabolism [183]. Additionally, Bmal1 is essential in adipogenesis; fibroblasts from Bmal1 knockout mice do not differentiate into adipocytes [184]. Consequently, understanding circadian–lipid interactions provides insights into the mechanisms underlying neurodegeneration and may offer potential targets for intervention.

A reciprocal relationship exists between circadian rhythms and lipid metabolism; a high-fat diet lengthens the intrinsic period of locomotor activity, disrupts feeding rhythms, and lowers the amplitude and shifts the phase of metabolic gene expression cycles across the liver, adipose tissue, and hypothalamus [185,186]. Similarly, genetically obese ob/ob and db/db mice exhibit altered circadian rhythms in activity and sleep–wake patterns [187]. Additionally, the metabolic hormone glucocorticoid, which has roles in processes such as gluconeogenesis, also entrains peripheral clocks and upregulates the expression of key clock genes such as Per1 and Per2 [188]. Furthermore, a study revealed sex-specific impacts of circadian desynchronization on metabolism in 14 young healthy adults [189]. Females with circadian misalignment showed increased energy expenditure, lipid oxidation, and hormonal changes compared with normal circadian conditions, while males had stable energy metabolism but elevated leptin under circadian misalignment [189]. Evidence from alpha-synuclein-overexpressing (ASO) mice showing age-dependent reductions in circadian patterns where daytime SCN neuron firing rates were reduced [190,191]. Also, circadian disruption accelerated nitrated-αSyn pathology spread, dopaminergic neuron loss, nitrative stress, and neuroinflammation [190,191]. Therefore, circadian genes have fundamental roles in lipid metabolism, which is closely associated with neurological health and the progression of neurodegenerative disorders.

Disruptions in circadian rhythms—because of factors such as irregular sleep patterns or shift work—can deregulate lipid metabolism and lead to abnormal lipid accumulation, oxidative stress, and neuroinflammation, which have been implicated in neurodegenerative diseases such as HD, AD, and PD. Disruptions in circadian clock genes contribute to lipid imbalances that increase oxidative stress and neuroinflammation—key factors in conditions such as HD, AD, and PD.

### 6.1. Huntington Disease (HD)

HD has been associated with substantial gray matter loss in the hypothalamus in the premanifest stage, thus leading to decreased habitual sleep efficiency and increased sleep arousal [192]. The circadian rhythm is regulated by the SCN within the hypothalamus, which controls melatonin production in the pineal gland. Alterations in melatonin secretion have been observed in patients with HD in various disease stages [193]. Whereas daytime melatonin levels have been found to be similar between patients with HD and controls, the evening rise in melatonin is delayed in patients with HD [194]. A more recent study has found lower 24 h average plasma melatonin levels, a flattened circadian rhythm of melatonin secretion, and higher variability in melatonin onset time in patients with premanifest or moderate HD than controls [195].

In transgenic R6/2 mice, a model for HD, circadian abnormalities have been observed, involving increased daytime activity and decreased nighttime activity [196]. This disruption of the sleep–wake cycle worsens with disease progression and eventually leads to complete behavioral disruption. These disturbances might stem from impaired regulation of the circadian rhythm by the SCN through clock genes. In R6/2 mice, abnormal expression of the clock genes Per2 and Bmal1 in the SCN, striatum, and motor cortex has been observed [196]. These findings suggest that SCN activity abnormalities associated with sleep disturbances might also occur in patients with HD. Normalizing circadian rhythms has been found to slow cognitive decline in HD animal models and potentially improve the quality of life in patients with HD [197]. Limited research has quantified clock gene expression in HD models. Studies in *Drosophila* have indicated that mutant huntingtin disrupts clock gene expression and leads to irregular sleep patterns [198]. Specifically, mutant huntingtin suppresses the expression of key circadian regulators—Per, timeless (tim), vrille (vri), par domain protein 1 (Pdp1), and clockwork orange (cwo)—during both dawn and dusk. These alterations in gene expression might be associated with the accumulation of mutant huntingtin in neural tissues, including the hypothalamus. Diminished expression levels of Per, tim, and vri have been associated with delayed nighttime sleep and influences on downstream circadian-regulated genes, thus potentially affecting sleep behavior at the organismal level in drosophila [199]. Furthermore, recent findings have indicated altered expression of clock genes, involving diminished expression of Per, tim, Clock, and cryptochrome, in an HD *Drosophila* model [198,199,200].

Interest in cholesterol homeostasis in HD has recently increased. Initial studies in small patient cohorts have found no significant lipid abnormalities, including cholesterol levels, in the plasma. However, one study reported that fibroblasts from patients with HD have been found to show no alterations in cholesterol content or in the activity of 3-hydroxy-3-methylglutaryl-CoA reductase (HMG-CoAR), the key enzyme in the sterol synthesis pathway in fibroblasts cells [201], suggesting the brain cell-specific function in regulating cholesterol levels in HD. Recent studies have indicated impaired cholesterol homeostasis in HD, in contrast to earlier findings. Patients with late-stage HD show diminished blood cholesterol levels, although whether the effects are due to the Huntingtin mutation or metabolic changes remains unclear. Because direct measurement of brain cholesterol in living individuals is impractical, most data come from cell lines and postmortem tissues. In a mutant-huntingtin-expressing cell line, diminished mRNA levels of cholesterol biosynthetic genes, including HMG-CoAR and 7-DHC reductase, have been observed [202]. Additionally, fibroblasts and postmortem striatal and cortical tissues from patients with HD exhibit diminished mRNA levels of these enzymes and de novo cholesterol synthesis [203]. Notably, one study reported elevated cholesterol content in the caudate nucleus in patients with HD, despite overall diminished sterol biosynthesis in the brain [204].

The elevated cholesterol content in HD, despite diminished synthesis, might be due to two mechanisms: decreased conversion of cholesterol to 24-OHC and enhanced uptake of plasma cholesterol. Whether cholesterol uptake from the plasma is altered in patients with HD remains unclear. Plasma levels of 24-OHC, generated by the enzyme CYP46, predominantly in neurons, are used to estimate brain cholesterol turnover. Unlike AD and multiple sclerosis, wherein elevated plasma 24-OHC indicates increased neuronal cholesterol catabolism, HD is associated with diminished plasma 24-OHC levels, possibly because of decreased neuronal volume and turnover; therefore, the diminished 24-OHC production in HD is probably due to inhibition of cholesterol excretion to maintain brain cholesterol homeostasis [205].

**Figure 3:**
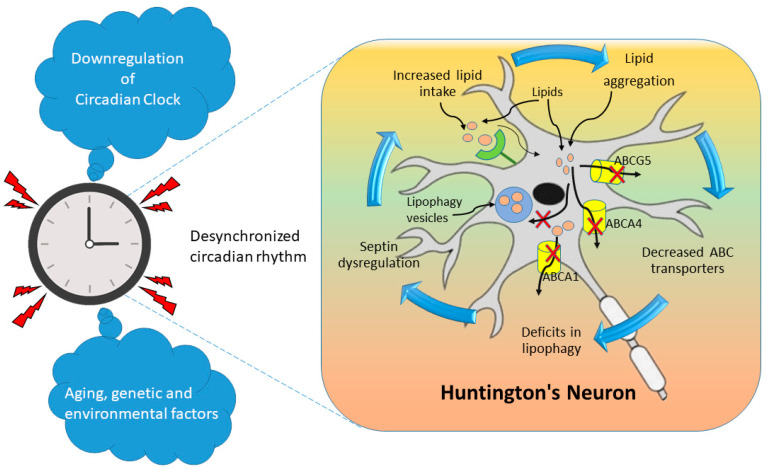
Dysregulated circadian rhythm and lipid metabolism drive neuronal insult in Huntington’s disease. Environmental factors downregulate circadian genes, thereby disrupting neuronal lipid homeostasis. This disturbance promotes increased lipid uptake by neurons, and results in lipid accumulation and subsequent downregulation of ABC transporters, including ABCA1, ABCA4, and ABCG5, which are critical for phospholipid and cholesterol transport. Diminished ABC transporter activity exacerbates lipid aggregation, impairs lipophagy, and hinders the clearance of excess lipids. This lipid overload contributes to septin dysregulation, which in turn impairs nerve conduction, and ultimately triggers neuroinflammation and neuronal degeneration associated with HD.

In summary, HD disrupts circadian rhythms and cholesterol homeostasis, both of which are critical to neurological health. A schematic diagram of pathways of neuronal degeneration due to deregulated lipid metabolism and circadian rhythm is provided in Figure 3. Altered melatonin secretion patterns and clock gene expression in HD suggest that circadian dysregulation might contribute to sleep disturbances and behavioral symptoms. Studies in HD highlight progressive disruption of sleep–wake cycles, possibly because of damage in the hypothalamus and SCN. Additionally, research has indicated impaired cholesterol synthesis and altered homeostasis in HD, particularly in the brain, despite increased cholesterol accumulation in specific regions [202,206]. These findings suggest that restoring circadian and cholesterol balance might help alleviate cognitive and behavioral issues in HD. However, how circadian clock genes regulate cholesterol metabolism remains incompletely understood in HD.

### 6.2. Alzheimer’s Disease (AD)

**Figure 4:**
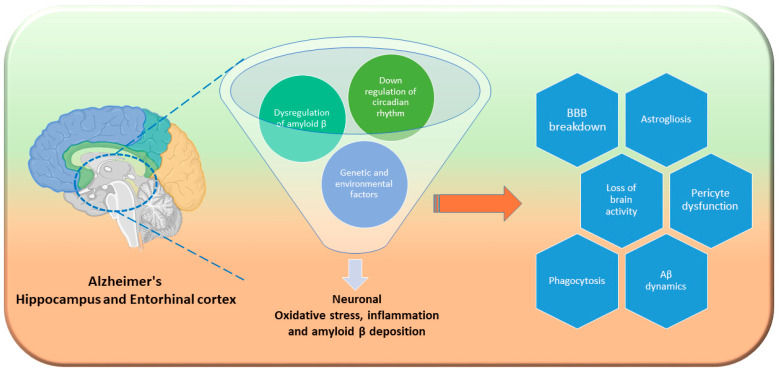
Hippocampal and entorhinal cortex insult in Alzheimer’s disease neurodegeneration. Primarily, the hippocampus and entorhinal cortex two are key regions for learning, memory, and spatial orientation. Genetic and environmental factors compromise circadian rhythm, followed by lipid metabolism. Loss of central circadian rhythms disrupts amyloid β fluid oscillations, speeding up amyloid plaque buildup. For example, downregulation of Bmal1 in the brain parenchyma elevates ApoE expression and encourages fibrillar plaque buildup [55]. Combined with Aβ plaque buildup and tau tangles, circadian disruption worsens neuroinflammation and impairs synaptic plasticity [212]. Subsequently, abnormal autophagy leads to BBB breakdown, and a loss of pericyte functioning aggravates the condition [215]. As circadian rhythm disturbances compound this molecular damage, neurodegeneration accelerates and drives AD progression.

The exact cause of AD remains unclear, although older age is a significant risk factor. The strongest genetic contributors are autosomal dominant genes involved in Aβ processing, including presenilin-1 (PSEN1), presenilin-2 (PSEN2), and amyloid precursor protein (APP); however, these account for only 1–5% of cases [207]. The sporadic form, which makes up most AD cases, involves complex interactions between genetic and environmental factors. The ApoEε4 gene might increase AD susceptibility and is associated with 15–20% of cases. Studies indicate that Abca7 also models APP processing and inhibits Aβ production in neurons, playing an important role in the development of AD [208]. Modifiable protective factors include higher education, social and physical activity, mentally stimulating tasks, moderate alcohol consumption, and omega-3 FA intake [209]. By contrast, midlife metabolic syndrome, diabetes, smoking, cerebrovascular disease, nutrient deficiencies, head injuries, and toxin exposure elevate AD risk, and some factors are age specific. Notably, lowering blood pressure, the body mass index, or cholesterol levels in older age correlates with higher AD incidence [210]. Several processes implicated in AD pathogenesis are interconnected with the circadian system, thereby suggesting potential shared mechanisms (Figure 4). However, the current data are inadequate to reveal the clinical significance of these theoretical connections, because of insufficiently rigorous clinical research and the use of transgenic animal models, which might not accurately represent the sporadic form of the disease.

Circadian rhythms and the sleep–wake cycle regulate Aβ levels. Both wild-type and Tg2576 mice exhibit a diurnal pattern of Aβ that peaks during wakefulness [211]. Similarly, human cerebrospinal fluid samples have shown a 25% variation in Aβ levels over 36 h, with higher concentrations during wakefulness. Aβ in the brain interstitial fluid is positively correlated with wakefulness and negatively correlated with non-REM sleep [212]. The CNS’s glymphatic system, which facilitates the clearance of metabolites including Aβ, increases the interstitial fluid space during sleep and enhances glymphatic flow. Sleep deprivation can increase Aβ plaques in transgenic mice and has been found to be detrimental to health in humans, as demonstrated by the substantial risks of fatal familial insomnia [212]. Aged mice show a 40% decrease in glymphatic flow, aligning with increased AD risk. In humans, cerebrospinal fluid Aβ amplitude is highest in healthy adults (18–60 years) and decreases significantly in older adults, particularly those with amyloid deposition detected by Pittsburgh compound B (PiB)-PET [213].

AD is associated with altered lipid composition in the brain, thereby affecting oligodendrocyte function and myelin integrity. These findings suggest that lipid metabolism is disrupted in AD, particularly in myelin and oligodendrocyte lineage cells, which are sensitive to lipid changes. Studies have indicated white matter abnormalities and oligodendrocyte dysfunction in brains with AD [214]. Further research is needed to explore the connections among altered brain lipid metabolism, oligodendrocyte dysfunction, and white matter abnormalities in AD. Additionally, obesity, metabolic syndrome, and CVD are known AD risk factors and are associated with brain atrophy. Investigating the roles of specific dietary lipids in ameliorating AD pathology and understanding peripheral dietary contributions will be important avenues for future research. Evidence from clinical and animal studies indicates a complex relationship between circadian rhythms and AD [212]. Although the etiology of AD is not fully understood, circadian rhythms interact with various systems and risk factors associated with AD development and progression. This interaction might provide a promising target for prevention and treatment. The relationship appears to be bi-directional, such that circadian interventions might potentially influence disease progression, provide symptomatic relief, and decrease socioeconomic effects. As precision medicine advances, a deeper understanding of the circadian rhythm-AD relationship may lead to more effective, personalized treatments [139].

### 6.3. Parkinson’s Disease (PD)

Studies in animal and cell models have shown a strong link between PD and cholesterol metabolism. The cholesterol biosynthesis pathway regulates PRKN (Parkin), and cholesterol uptake has been found to be affected in PRKN mutant models [216]. DJ-1 knockout models exhibit low cellular cholesterol and impaired endocytosis, which are restored by the addition of membrane cholesterol. By contrast, glucosylceramidase beta 1 (GBA) and PRKN knockout cells show elevated cholesterol [217,218]; the N370S GBA mutation causes lysosomal accumulation, and leucine-rich repeat kinase 2 (LRRK20) knockout rats display elevated serum cholesterol [219,220]. These findings suggest that cholesterol biosynthesis is disrupted in PD, and variations are observed depending on the genetic cause, thereby explaining observed inconsistencies among patients with PD.

Alpha-synuclein (α-synuclein) interacts with cholesterol and cholesterol-containing vesicles, thus potentially influencing its binding to charge-neutral membranes. This interaction is associated with α-synuclein accumulation and aggregation, which are critical for pore formation [221]. Low cholesterol levels decrease α-synuclein accumulation and synaptic damage, whereas high cholesterol levels exacerbate α-synuclein-associated pathology [222]. Additionally, α-synuclein enhances cholesterol efflux, disrupts cholesterol in lipid rafts, and increases oxidative cholesterol metabolites [223]. Mice overexpressing A53T-α-synuclein show elevated serum cholesterol, whereas those with wild-type α-synuclein have upregulated cholesterol biosynthesis genes in dopaminergic neurons from the substantia nigra [222,224]. These findings indicate a reciprocal relationship between α-synuclein and cholesterol metabolism.

**Figure 5:**
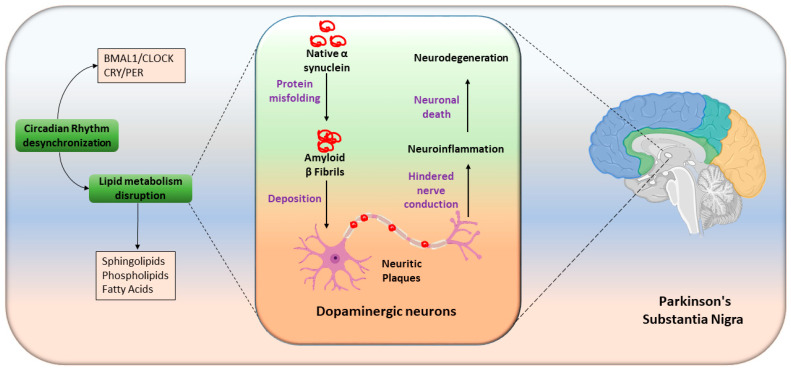
Pathophysiology of Parkinson’s disease: Under physiological conditions, α-synuclein exists as a soluble random coil. Pathological conditions are caused by abnormal lipid metabolism and desynchronized circadian rhythm. α-synuclein is misfolded, thus forming toxic dimers, trimers, and oligomers. These misfolded forms aggregate into protofibrils, intermediates, and amyloid fibrils. Aggregates form Lewy bodies or Lewy neurites and deposited over dopaminergic neurons and lead to neuroinflammation, neuronal death, and subsequently degradation of the substabtia nigra.

Increased cholesterol can decrease cell death and enhance the presynaptic dopaminergic phenotype by elevating tyrosine hydroxylase (TH) and vesicular monoamine transporter 2 (VMAT2) expression in SH-SY5Y cells, as well as increasing ligand binding of dopamine transporters (DAT) and VMAT2 in rat and monkey brains [225]. Additionally, hypercholesterolemia is associated with dopamine neuronal loss and oxidative stress in the substantia nigra and striatum [226], thus leading to motor impairment. Additionally, cholesterol treatment in MPP^+^-treated SH-SY5Y cells decreases cell viability [227]; therefore, the effects of cholesterol on PD might be concentration-dependent [226,228]. Body weight loss is common in patients with PD and correlates with faster disease progression and poorer outcomes [229]. Weight regulation involves energy balance, which is influenced by circadian-controlled energy expenditure, suggesting PD is very closely related with a dysregulated circadian clock [230]. Circadian disruptions in PD, including altered sleep–wake cycles and excessive daytime sleepiness, are well documented. Molecular studies have shown that patients with PD have diminished nighttime expression of BMAL1, a key circadian regulator, and this expression correlates with disease severity and is unaffected by medication. Increased early morning expression of PER2 and REV-ERBα, along with a generally dampened BMAL1 profile, further indicate disrupted circadian rhythms in PD. Dopaminergic neuron insult is summarized in Figure 5 on the basis of known findings. Dopamine serves as a circadian input by stimulating CLOCK transcription through dopamine receptors in the nervous system, including the SCN. However, further studies are needed to fully understand clock gene regulation in PD.

## 7. Future Directions and Perspectives

In conclusion, circadian rhythms are fundamental in regulating numerous physiological processes and metabolic functions. The SCN, along with core clock genes such as Clock, Bmal1, Per, and Cry, operates as the central pacemaker, thereby maintaining homeostasis by coordinating these rhythms with environmental cues. Disruptions in circadian rhythms can have profound health consequences that have been linked to neurodegenerative diseases. Emerging evidence highlights critical connections between circadian rhythm dysregulation and lipid metabolism abnormalities in the brain. This association exacerbates lipid dysregulation and increases oxidative stress and the accumulation of neurotoxic lipid derivatives, such as ApoE, α-synuclein, Lewy bodies, and Aβ fibrils. These derivatives are deposited in various regions of functional neurons, thus forming neuritic plaques and restricting nerve conduction. Subsequently, neuroinflammation and neurodegeneration occur in various regions of the brain implicated in neuronal disorders including AD, HD, and PD.

This pathogenesis is not limited to the formation of neuronal plaques but also includes dysregulation of ABC transporters (such as Abca1, Abcg1, Abcg4, and Abca7), Npc1 and Npc2, microglial functions, and proteins (such as septins) associated with the proliferation of neural progenitor cells, neuritogenesis, and synapse formation. Some TG- or FA-associated genes, such as Dgat1, Dgat2, Cd36, Mtp, and apolipoproteins, are expressed in the SCN and show expression fluctuations over a 24 h period. These genes therefore may play critical roles in circadian rhythm associated neurodegeneration, although no strong evidence has been reported, and further investigation is necessary. Circadian rhythms can influence lipid metabolism; by contrast, the transport and availability of lipids can influence circadian gene expression and neuronal activity. Therefore, these transporters and enzyme proteins might be crucial in linking lipid homeostasis with circadian regulation, ultimately supporting neuronal health and function throughout the day–night cycle. Further research is needed to elucidate the specific mechanisms through which these transporters interact with circadian rhythms.

Future research should focus on elucidating the mechanisms through which circadian disruptions influence lipid regulatory pathways in the brain, as well as exploring therapeutic strategies that target circadian and lipid homeostasis. These insights might lead to novel interventions aimed at mitigating the effects of circadian rhythm disturbances on neurological health and preventing the onset or progression of neurodegenerative diseases.

## Data Availability

Not applicable.

## Abbreviations

Aβ—Amyloid beta, AD—Alzheimer’s disease, ABCA1—ATP-Binding Cassette Sub-Family A Member 1, ABCG1—ATP-Binding Cassette Sub-Family G Member 1, ACAT—Acyl-CoA: Cholesterol Acyltransferase, Apo—Apolipoprotein, APP—Amyloid Precursor Protein, BACE1—Beta-Site Amyloid Precursor Protein-Cleaving Enzyme 1, BBB—blood–brain barrier, BMAL1—Brain and Muscle Arnt-Like Protein 1, ChIP-Seq—Chromatin Immunoprecipitation Sequencing, CK1δ/ε—Casein Kinase 1 Delta/Epsilon, CLOCK—Circadian Locomotor Output Cycles Kaput, CRY—Cryptochrome, CNS—Central Nervous System, CYP46—cholesterol 24-hydroxylase, DBP—D-site Albumin Binding Protein, DEC1—Basic Helix–Loop–Helix Family Member E40, DEC2—Basic Helix–Loop–Helix Family Member E41, DHA—Docosahexaenoic Acid, ER—Endoplasmic Reticulum, FAs—Fatty Acids, FAS—Fatty Acid Synthase, GR—Glucocorticoid Receptor, HC—hydroxycholesterol, HD—Huntington’s Disease, HDL—High-Density Lipoprotein, IFN-γ—Interferon Gamma, IL—Interleukin, IGL—Intergeniculate Nucleus, LPS—Lipopolysaccharide, LDL—Low-Density Lipoprotein, Mfsd2a—Major Facilitator Superfamily Domain-Containing Protein 2A, MMP—Matrix Metalloproteinase, NF-κB—Nuclear Factor Kappa B, NLRP3—NOD-Like Receptor Protein 3, NPC—Niemann-Pick C1-Like Protein, PER—Period, PLC—Phospholipase C, PLC-γ1—Phospholipase C Gamma 1, PVN—Paraventricular Nucleus, PD—Parkinson Diseases, PP1—Protein Phosphatase 1, REV-ERBα—Nuclear Receptor Subfamily 1 Group D Member 1, REM—Rapid Eye Movement (sleep), ROR—Retinoid-Related Orphan Receptor, SCN—Suprachiasmatic Nucleus, SREBP—Sterol Regulatory Element-Binding Protein, SNPs—Single-Nucleotide Polymorphisms, TGF-β—Transforming Growth Factor Beta, TLR—Toll-Like Receptor, TNF-α—Tumor Necrosis Factor Alpha, TTO—Transcription Translation Oscillating Loop, TG—Triglycerides, TRL—Triglyceride-Rich Lipoprotein, VLDL—Very-Low-Density Lipoprotein.
